# Supplementary material for: Light Regulation of Two New Manganese Peroxidase-Encoding Genes in Trametes polyzona KU-RNW027
Source: Microorganisms. 2020 Jun 5;8(6):852. doi: 10.3390/microorganisms8060852 (PMC7355636; doi:10.3390/microorganisms8060852)
Supplement: Supplementary file 1 [file microorganisms-08-00852-s001.zip › Table S1-edited.docx]

Table S1. Oligonucleotide primers.

| **Primer** | **Oligonucleotide sequence** | **Purpose** |
| --- | --- | --- |
| MnPf | 5’-CCTTCCACGACGCSATCGG-3’ | Forward degenerated primer for *mnp* |
| MnPr | 5’-GCASGCCTGCTCSACGTC-3’ | Reverse degenerated primer for *mnp* |
| Lacf | 5’-CACTGGCAYGGBTTYTTCC-3’ | Forward degenerated primer for *lac* |
| Lacr | 5’-GGAAGTCGATGTGGCAGTG-3’ | Reverse degenerated primer for *lac* |
| M1_1 | 5’-GGTTCTGCTGTATGTCATCC-3’ | Nested primer for 5’-RACE for *mnp1* |
| M1_2 | 5’-CGAAGATGGCGATGGAGC-3’ | Primer for 5’-RACE for *mnp1* |
| M1_3 | 5’-CCTTCCAGTCCGACTTTGAGC-3’ | Primer for 3’-RACE for *mnp1* |
| M2_1 | 5’-GCGTGGAAGTTGGTCTCG-3’ | Nested primer for 5’-RACE for *mnp2* |
| M2_2 | 5’-GCAGTGGTGATGTTGTGGC-3’ | Primer for 5’-RACE for *mnp2* |
| M2_3 | 5’-CAACAACCAGGCGAAGATGC-3’ | Primer for 3’-RACE for *mnp2* |
| L1 | 5’-GGCACTGGTTGATGAACGC-3’ | Nested primer for 5’-RACE for *lac1* |
| L2 | 5’-GGAGGTCGTACAGGAAGGC-3’ | Primer for 5’-RACE for *lac1* |
| L3 | 5’-CATCTCCACGGTCACACTTTCG-3’ | Primer for 3’-RACE for *lac1* |
| iMnP1-1 | 5’-CATCCCGTCAGCATTCAC-3’ | Inverse PCR |
| iMnP1-2 | 5’-CTTCGTCAGTGCGTATCCTC-3’ | Inverse PCR |
| iMnP2-1 | 5’-CGGAATTGTGACAAGGAGC-3’ | Inverse PCR |
| iMnP2-2 | 5’-CCTGTCATTCTCAACACACG-3’ | Inverse PCR |
| iLac1 | 5’-GCGTTCATCAACCAGTGC-3’ | Inverse PCR |
| iLac2 | 5’-CATCTCCACGGTCACACTTTCG-3’ | Inverse PCR |
