# Supplementary material for: Light Regulation of Two New Manganese Peroxidase-Encoding Genes in Trametes polyzona KU-RNW027
Source: Microorganisms. 2020 Jun 5;8(6):852. doi: 10.3390/microorganisms8060852 (PMC7355636; doi:10.3390/microorganisms8060852)
Supplement: Supplementary file 1 [file microorganisms-08-00852-s001.zip › Table S2-edited.docx]

Table S2. Oligonucleotide primers for RT-qPCR.

| **Primer** | **Oligonucleotide sequence** | **Amplicon (bp)** |
| --- | --- | --- |
| MnP1f | 5’-CTAAACGACGAACACCTGC-3’ | 130 |
| MnP1r | 5’-GGAACAAAGCAGGGAGAGC-3’ |  |
| MnP2f | 5’-CTCAGCCATCTCCTGTAACG-3’ | 147 |
| MnP2r | 5’-ACAGGCAGCGTTAGAAGC-3’ |  |
| Lac1f | 5’-CATCTCCACGGTCACACTTTCG-3’ | 222 |
| Lac1r | 5’-AGTGTCCTCAGCCATAACG-3’ |  |
| rDNAf | 5’-CCGTTCTTAGTTGGTGGAG-3’ | 211 |
| rDNAr | 5’-ACTCGCTGGCTCTGTCAG-3’ |  |
